# Modified Combined Anterior Cruciate Ligament and Anterolateral Ligament Reconstruction in 291 High-Level Athletes: Clinical Outcomes at Minimum 2.5-Year Follow-Up

**DOI:** 10.3390/medicina61101762

**Published:** 2025-09-29

**Authors:** Tomislav Kottek, Stjepan Bulat, Goran Vrgoč, Alan Ivković, Frane Bukvić, Joško Jeličić, Saša Janković

**Affiliations:** 1 Department of Orthopaedics and Traumatology, Clinical Hospital “Sveti Duh”, 10000 Zagreb, Croatia; kottek34@gmail.com (T.K.); bulatstjepan@gmail.com (S.B.); alan.ivkovic@gmail.com (A.I.); frane.bukvic@gmail.com (F.B.); josko.jelicic@gmail.com (J.J.); sjanko@kif.unizg.hr (S.J.); 2 University of Zagreb Faculty of Kinesiology, 10000 Zagreb, Croatia; 3 University of Zagreb School of Medicine, 10000 Zagreb, Croatia

**Keywords:** anterior cruciate ligament, anterolateral ligament, anterior cruciate ligament reconstruction, athletes, return to sport

## Abstract

*Background and Objectives*: Combined anterior cruciate ligament (ACL) and anterolateral ligament (ALL) reconstruction has been advocated to improve rotational stability and reduce graft failure in high-risk athletes. We aimed to evaluate the mid-term functional outcomes of a modified combined ACL and ALL reconstruction technique using hamstring tendon autografts developed at our institution. *Materials and Methods*: We retrospectively reviewed 395 patients who underwent combined ACL and ALL reconstruction between 2018 and 2022. Of these, 291 patients (73.6%) completed the minimum follow-up of 2.5 years and were included in the analysis. Primary outcomes were graft rerupture and return to sport (RTS) at the pre-injury level. Secondary outcomes included graft survival, a change in Tegner score from pre-injury to follow-up and complications. *Results*: The cohort consisted of 219 males (75.3%) and 72 females (24.7%), with a mean age of 20.6 ± 4.0 years (range 14–35). Eleven patients experienced graft rerupture, yielding a rate of 3.78% (95% CI, 2.1–6.6). At final follow-up, 220 patients (75.6%; 95% CI, 70.4–80.2) returned to their pre-injury level of sport performance. The mean Tegner activity score decreased from 7.9 ± 1.4 preoperatively to 7.2 ± 1.8 postoperatively (paired *t*-test, *p* < 0.0001; Wilcoxon signed-rank test, *p* < 0.0001). Postoperative complications occurred in 18 patients (6.2%), the majority of which related to meniscal re-ruptures. *Conclusions*: Our modified combined ACL and ALL reconstruction technique demonstrated excellent mid-term results in a high-risk athletic population, with low rerupture rates and high RTS rates, while also being a safe procedure without significant complications. These findings support the use of this technique in young and professional athletes where rotational stability is necessary.

## 1. Introduction

Anterior cruciate ligament (ACL) injury is one of the most common knee injuries in young, active individuals, with an increasing incidence among athletes participating in pivoting sports, particularly those aged 14–25 years [1].

Although modern arthroscopic ACL reconstruction effectively restores anterior–posterior knee stability, residual rotational instability remains a frequent postoperative concern. This instability is associated with suboptimal functional outcomes, high rerupture rates, and reduced rates of return to sport [2]. To address these limitations, various lateral extra-articular procedures (LEAPs) have been developed to enhance rotational stability and improve postoperative results. The most frequently employed techniques include Lemaire’s lateral extra-articular tenodesis (LET), the MacIntosh procedure, Ellison’s distal iliotibial tract transfer, the Marcacci/Zaffagnini technique, and anterolateral ligament (ALL) reconstruction [3].

Although the anterolateral structures of the knee have been studied and described since the 19th century [4], ALL has regained significant attention in the orthopedic community over the past decade. In 2013, Claes et al. [5] clearly defined its anatomy through meticulous cadaveric dissection, and ALL was identified as a distinct ligament originating from the lateral femoral epicondyle and inserting slightly posterior to Gerdy’s tubercle on the proximal tibia. Subsequent dissections suggested that its femoral origin is located slightly posterior and proximal to the lateral femoral epicondyle [6,7]: a finding crucial for maintaining its isometry—remaining taut in extension and slack at 90° of flexion. Biomechanically, this makes the ALL an important stabilizer of internal tibial rotation, particularly from 30° of knee flexion onwards [8,9,10]. Visualization of the ALL has been reported arthroscopically [11], by magnetic resonance imaging [12,13] and ultrasonography [14]. Despite these insights, in the years following its “rediscovery”, ALL became the subject of considerable debate and controversy, with some questioning not only its role in rotational knee stability but even its existence [15,16,17,18,19,20].

Based on the available evidence and their own clinical experience, Bertrand Sonnery-Cottet and colleagues developed a combined anatomic ACL and ALL reconstruction technique using hamstring tendon autografts, aiming to improve both subjective and objective outcomes [21]. Clinical studies have since demonstrated that, in high-risk populations such as young athletes in pivoting sports, this combined reconstruction significantly lowers rerupture rates and increases the likelihood of returning to preinjury levels of sport compared with isolated ACL reconstruction [3,22,23]. Importantly, the procedure has proven safe, does not delay recovery, reduces revision rates, and may protect repaired medial menisci [24,25,26,27,28,29].

Inspired by this approach, the orthopedic team at Clinical Hospital “Sveti Duh” developed a modified combined ACL and ALL reconstruction technique, first described and published in 2021 [30]. The technique was designed to preserve bone stock, optimize graft incorporation, and allow intraoperative adjustment of graft tension—features intended to optimize stability and long-term outcomes compared with established techniques. The present study retrospectively evaluates the clinical outcomes of this technique in a large cohort of high-level patients with a minimum follow-up of two and a half years. Our focus on high-level athletes, referring primarily to young individuals engaged in pivoting or contact sports, stems from the fact that this population is at great risk for ACL injury and rerupture, and thus requires surgical strategies that maximize graft survival and enable long-term continuation of sport at demanding levels. We hypothesized that our modified combined ACL and ALL reconstruction technique would result in a low graft rerupture rate and high rates of return to pre-injury sport levels in a young, high-risk athletic population.

## 2. Materials and Methods

### 2.1. Study Design and Setting

This was a single-center retrospective cohort study of patients undergoing combined anterior cruciate ligament (ACL) and anterolateral ligament (ALL) reconstruction between January 2018 and December 2022. All procedures were performed by three fellowship-trained orthopedic sports medicine surgeons at Clinical Hospital “Sveti Duh”. The study was approved by the institutional review board, and all participants provided informed consent.

### 2.2. Eligibility Criteria

Indications for combined ACL and ALL reconstruction were: participation in high-demand contact or pivoting sports, positive pivot-shift test (grade 2 or 3), or age < 20 years, regardless of the level of sports activity. Contraindications included: a concomitant medial collateral ligament (MCL), lateral collateral ligament (LCL) or posterior cruciate ligament (PCL) rupture, hamstring insufficiency, insufficient gracilis tendon size, and a lack of knowledge about ALL anatomy in the treatment team. Patients who underwent revision ACL reconstruction surgery were not included in the study.

### 2.3. Surgical Technique

Our modified technique has previously been described in more detail [30] and is summarized here.

Two convergent 4.5-mm tunnels were drilled in the proximal tibia for later ALL graft passage. Through a vertical incision 1 cm medial to the tibial tuberosity, the semitendinosus and gracilis tendons were harvested, whipstitched, and prepared. The semitendinosus was tripled over a TightRope (Arthrex, Naples, FL, USA) to a length of 8–8.5 cm; the gracilis was placed inside and secured with multiple No. 2-0 Vicryl sutures for tubularization. A No. 2 FiberWire (Arthrex, Naples, FL, USA) was attached to the femoral end.

The femoral tunnel was drilled outside-in to match the graft size. A tibial guide was set at 55°, and retro-drilling of the tibial tunnel was performed to a depth of 35 mm, corresponding to the graft diameter, preserving the ACL remnant and tibial bone stock.

The TightRope and graft were passed through the iliotibial band (ITB) and femoral tunnel into the tibial tunnel, but only up to 30 mm of the graft on the tibial side, which had been pre-marked, leaving an additional 5 mm for subsequent tensioning of the graft with the TightRope system. With the knee at 90°, the TightRope was tightened on the tibial side; at 20°, the graft was fixed with a 23-mm outside-in bioabsorbable interference screw. Graft tension was checked through the full range of motion, and additional tension, if needed, is secured through the TightRope system. The gracilis strand was passed under the ITB to the tibial tunnel, shuttled proximally, and tied with FiberWire in extension and neutral rotation at the place of ALL origin.

### 2.4. Rehabilitation Protocol

Our patients did not follow a standardized rehabilitation program, as postoperative rehabilitation was performed at various institutions according to patient choice and availability. Nevertheless, several key elements were consistently emphasized. Immediate full weight bearing without a brace was permitted, accompanied by progressive range-of-motion exercises. Early rehabilitation focused on regaining full extension and activating the quadriceps, particularly the vastus medialis, while progression was guided by achievement of functional milestones rather than fixed timelines.

### 2.5. Outcome Measures

Primary outcomes were ACL graft rerupture rate and return to sport (RTS) at the same pre-injury level, assessed using the Tegner score and patient self-report. Secondary outcomes included graft survival, changes in Tegner score from pre-injury to follow-up and complications.

### 2.6. Data Collection

Demographic, injury-related, and surgical data were collected retrospectively from operative records. Follow-up data, including RTS status and Tegner scores, were obtained via standardized telephone interviews. Graft reruptures were confirmed by clinical examination, MRI, and/or revision surgery.

### 2.7. Statistical Analysis

Statistical analyses were performed using BM SPSS Statistics, Version 27.0 (IBM Corp., Armonk, NY, USA). Descriptive statistics (means, standard deviations, medians, and ranges) were used for demographic and clinical data. Rerupture and return-to-sport rates were expressed as percentages with 95% confidence intervals. Changes in Tegner activity scores from pre-injury to follow-up were analyzed using both paired *t*-test and Wilcoxon signed-rank test to account for non-normal distribution. Kaplan–Meier survival analysis was used to estimate graft survival over time. Normality of continuous variables was assessed using the Shapiro–Wilk test. A *p*-value of < 0.05 was considered statistically significant.

## 3. Results

A total of 395 patients underwent combined ACL and ALL reconstruction between 2018 and 2022. Of these, 291 patients (73.6%) completed the minimum follow-up of 2.5 years and were included in the final analysis. The mean follow-up duration was 58.2 ± 16.7 months (median, 57.7; range, 30–90 months). The cohort consisted of 219 males (75.3%) and 72 females (24.7%). The mean age at the time of surgery was 20.6 ± 4.0 years (median, 20; range, 14–35).

### 3.1. Rerupture Rate

**Figure 1:**
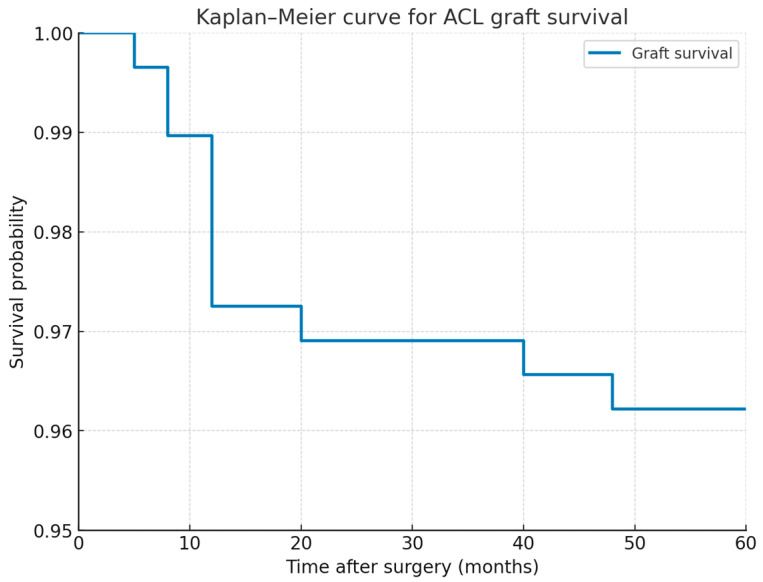
Survivorship data from Kaplan–Meier analysis.

Eleven patients experienced graft rerupture, yielding an overall rerupture rate of 3.78% (95% CI, 2.1–6.6). All cases were confirmed by clinical examination, MRI, and/or revision surgery. The median time to graft rerupture was 12 months (range, 5–48 months). The majority of reruptures (7/11; 63.6%) occurred within the first postoperative year, while 4 cases (36.4%) were late events occurring beyond 20 months after surgery. Figure 1 shows the survivorship data from Kaplan–Meier analysis, demonstrating a graft survival probability of 96.2% at 48 months.

### 3.2. Return to Sport

At final follow-up, 220 patients (75.6%; 95% CI, 70.4–80.2) successfully returned to sport at the same pre-injury level. This cohort included elite and high-demand athletes, particularly professional soccer players and participants in other pivoting or contact sports.

### 3.3. Tegner Activity Scale

The mean preoperative Tegner activity score was 7.9 ± 1.4, compared with 7.2 ± 1.8 postoperatively (mean difference −0.7; 95% CI –0.9 to –0.5; paired *t*-test, *p* < 0.0001; Wilcoxon signed-rank test, *p* < 0.0001). While this result suggests a statistically significant drop in Tegner activity from 7.9 to 7.2 (mean difference −0.7), this change is slightly below the commonly referenced MCID threshold for Tegner (~0.9 points), suggesting that although the change is measurable, it may not be clinically meaningful in many of our patients.

Preoperative and postoperative activity levels were assessed using the Tegner Activity Scale:

- Among 55 patients (14.0%) with a preoperative score of 10, 43 (78.2%) regained the same score postoperatively.
- Among 35 patients (8.9%) with a preoperative score of 9, 24 (68.6%) regained the same score.
- Among 153 patients (38.7%) with a preoperative score of 7 or 8, 128 (83.7%) regained the same score.

Overall, a high proportion of patients were able to return to their pre-injury activity level, with particularly favorable outcomes observed among those with Tegner scores of 7–8.

Among patients who returned to a lower activity level (n = 75), the majority experienced only a modest decline in Tegner score. Specifically, 13 patients out of 75 (17.3%) dropped by one level, 5 patients (6.7%) by two levels, and 31 patients (41.3%) by three levels. More pronounced decreases were less frequent, with 5 patients (6.7%) experiencing a four-level decline and 4 patients (5.3%) a five-level decline. Three patients reported lower postoperative Tegner scores due to unrelated health issues rather than knee function itself.

**Figure 2:**
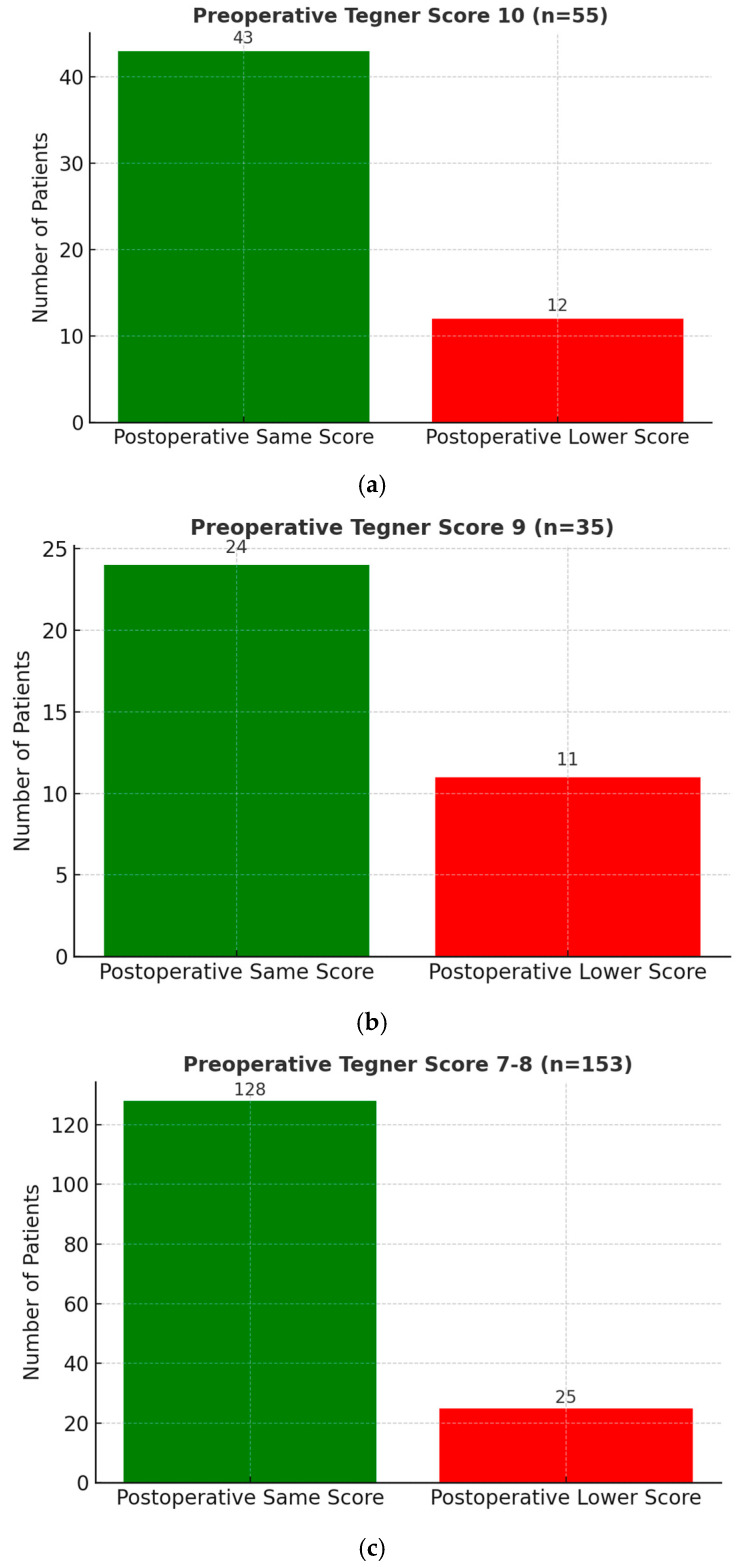
Recovery of preoperative Tegner scores: (**a**) Among 55 patients with a preoperative Tegner score of 10, 43 (78.2%) maintained the same score postoperatively, while 12 (21.8%) returned at a lower level. (**b**) Among 35 patients with a preoperative Tegner score of 9, 24 (68.6%) maintained the same score postoperatively, while 11 (31.4%) returned at a lower level. (**c**) Among 153 patients with a preoperative Tegner score of 7–8, 128 (83.7%) maintained the same score postoperatively, while 25 (16.3%) returned at a lower level.

Surgical outcomes are summarized in Table 1 and Figure 2.

### 3.4. Complications

**Figure 3:**
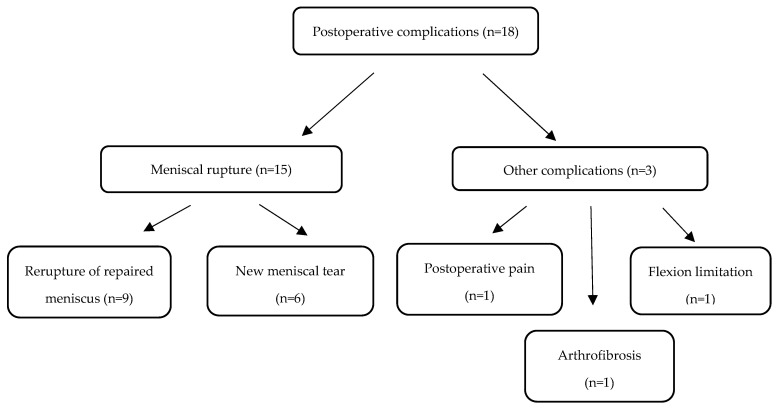
Postoperative complications reported.

Postoperative complications were reported by 18 out of 291 patients (6.2%). The majority of these (15/18, 83%) were related to meniscal rupture. Of these, 9 cases occurred in menisci that had been primarily repaired during the index procedure, while the remaining 6 cases represented new meniscal tears in previously untreated menisci. Other complications included one case (0.3%) of postoperative pain, one case (0.3%) of partial limitation of flexion, and one case (0.3%) of arthrofibrosis. No infections or thromboembolic events were observed. Complications are presented in Figure 3.

These outcomes, achieved in a cohort with one of the highest reported proportions of elite-level and high-demand athletes in the literature, represent favorable results for combined ACL and ALL reconstruction in such a challenging patient population. Comparable return-to-sport rates were observed across all Tegner subgroups, confirming that even top-level professional athletes were able to return to their pre-injury performance.

## 4. Discussion

This study demonstrates that our modified combined ACL and ALL reconstruction technique yields a low rerupture rate (3.78%) and a high rate of return to preinjury sport levels (75.6%) in a high-risk athletic population, with a minimum follow-up of two and a half years. Our findings align with numerous published studies, many of which also report superior outcomes for combined ACL and ALL reconstruction compared with the isolated ACL reconstruction.

In a prospective comparative study published in 2017, the SANTI Study Group was the first to highlight the clinical benefits of anatomic combined ACL and ALL reconstruction in a high-risk population of young athletes engaged in pivoting sports, comparing this approach with isolated ACL reconstruction using either a bone–patellar tendon–bone (BTB) graft or a quadrupled hamstring tendon graft. In their series, the rerupture rate in the combined reconstruction group was 4.13%, which was 2.5 times lower than in the BTB group and 3.1 times lower than in the quadrupled hamstring group. Furthermore, the proportion of athletes returning to the same sport level was 68.8%, exceeding the 59.9% observed in the quadrupled hamstring group [23]. Other authors have reported similar findings. Helito et al. demonstrated superior functional outcomes and lower rerupture rates using combined ACL and ALL reconstruction in patients with chronic ACL injury [31] and in those with generalized ligamentous laxity [32]. The SANTI Study Group also provided long-term results in a series with a mean follow-up of 104 months, showing significantly better graft survival and a lower reoperation rate compared with isolated ACL reconstruction [28].

Beyond its clinical efficacy, the technique has been shown to be safe, with no increased risk of complications or reoperations compared with isolated ACL reconstruction [27,33]. Moreover, in addition to protecting the ACL graft, ALL reconstruction has been reported to offer a protective effect on repaired medial menisci [24,34]. Patients treated with combined ACL and ALL reconstruction demonstrated higher meniscal repair survival (91.2% versus 83.8% survival rate at 36 months) and more than a twofold lower risk of medial meniscal repair failure compared with those who underwent isolated ACL reconstruction [24].

From a biomechanical perspective, the ALL serves as a key secondary stabilizer of internal tibial rotation, particularly beyond 30° of knee flexion, where standard ACL reconstruction alone may not provide sufficient rotational control [8]. In high-risk patients—such as young athletes in pivoting sports or those with generalized ligamentous laxity—residual rotational instability after isolated ACL reconstruction increases the load on the graft and may contribute to premature graft failure. Anatomical ALL reconstruction reduces the pivot-shift phenomenon and redistributes intra-articular forces, thereby creating a more stable mechanical environment for better ACL graft incorporation and maturation, all of which facilitate safer and faster return to sport [35,36]. Interestingly, long-term follow-up data have also helped to dispel earlier concerns that ALL reconstruction could predispose to lateral compartment osteoarthritis [37].

These advantages of combined ACL and ALL reconstruction are particularly relevant for young and professional athletes—a population in which outcomes following isolated ACL reconstruction remain suboptimal. In this group, rerupture rates have been reported between 18% and 28% [38,39]. Likewise, return-to-sport rates vary widely across studies, ranging from 83% of elite athletes resuming their preinjury sport performance level [40] to as few as 55% returning to competitive sport [41]. Several studies focusing specifically on professional athletes have confirmed excellent results. Rosenstiel et al. [42] reported, in a cohort of 70 professional athletes with a minimum follow-up of two years, a rerupture rate of 5.7% and a return-to-sport rate of 85.7%. Laboudie et al. [34] found, in athletes younger than 20 years, a rerupture rate of 5.8% and a return-to-sport rate of 52%, again with superior outcomes compared to isolated ACL reconstruction. In a cohort of 342 professional athletes with a mean follow-up of 100 months, the SANTI Study Group reported that isolated ACL reconstruction was associated with a more than twofold increased risk of graft rupture [HR 2.678] compared with combined ACL reconstruction with a lateral extra-articular procedure [43]. Of note, combined reconstruction has also been shown not to delay recovery or return-to-sport timelines—an important consideration for elite athletes [29].

When considering lateral extra-articular procedures, it is important to recognize that both LET and ALL reconstruction have been shown to safely improve rotational stability and reduce graft failure rates when performed correctly, with no conclusive evidence supporting the superiority of one technique over the other [44,45]. Anatomical ALL reconstruction aims to restore native ligament biomechanics with potentially less alteration of normal knee kinematics. Our approach follows this anatomic philosophy while incorporating specific technical modifications intended to further optimize graft performance.

We are encouraged that our results are comparable to those reported in other studies and even exceed those in some series. Our analysis demonstrated excellent graft survival, with 97.3% of grafts intact at 12 months and 96.2% at 48 months. These findings suggest that the risk of graft failure is highest during the early postoperative period, while long-term survival remains favorable. The overall complication rate of 6.2% in our series, where most complications were attributable to meniscal ruptures (5.2%) rather than graft-related issues, is consistent with previously published reports of combined ACL and ALL reconstruction [27]. The absence of severe complications such as infection or thromboembolic events highlights the favorable safety profile of our modified technique. Although the 75.6% rate of return to sport at the pre-injury level in our cohort is favorable, it is somewhat lower than in certain previously published series [23], which may reflect differences in surgical technique, the absence of a standardized rehabilitation protocol, and individual psychological or contextual factors influencing athletes’ decisions to return. Among the 75 patients who returned to a lower activity level, the majority experienced only a modest decline in Tegner score. Three patients reported lower postoperative Tegner scores due to unrelated health issues rather than knee function itself. Therefore, it is important to recognize that a decrease in Tegner activity score does not necessarily reflect knee-related limitations. Factors such as psychological barriers, other health conditions, or personal life circumstances may influence return-to-sport outcomes [46].

Our modified ACL–ALL reconstruction technique differs from the original combined reconstruction method [21] primarily in that the tibial tunnel is created by retrograde drilling, typically to a depth of approximately 35 mm. This preserves tibial bone stock, which may be considered an advantage. In addition, the tibial side of the graft is fixed using an adjustable suspensory fixation [ASF] device—specifically, a TightRope button. Colombet et al. reported improved graft incorporation and reduced tunnel widening with ASF compared with bioabsorbable interference screws, which may represent another potential advantage of our approach [47]. Moreover, after femoral fixation with an interference screw, our technique allows additional tensioning of the graft via the TightRope system before final fixation. In contrast, in the original method, graft tension cannot be modified once the femoral screw has been placed. This ability to fine-tune tension may represent one of the major technical advantages of our modification. On the other hand, we do not preserve the native tibial insertion of the semitendinosus tendon, which could be viewed as a limitation. Some studies have suggested that retaining the tendon’s tibial attachment may promote graft reinnervation and revascularization [48] and provide superior mechanical properties [49]. However, a recent meta-analysis found no significant differences in clinical outcomes—regarding stability, pain, or function—between grafts harvested with and without preserved tibial insertion [50]. Another potential drawback of our technique is the need for an orthopedic assistant familiar with the graft preparation process. Nevertheless, the ability to prepare the graft on the working station and create the bone tunnels simultaneously can shorten operative time.

Taken together, these technical modifications offer both theoretical and practical benefits, including preservation of tibial bone stock, the potential for improved graft incorporation and the ability to fine-tune graft tension intraoperatively. The modifications used in our technique may have contributed to the favorable outcomes observed; however, we interpret them as potential advantages rather than proven causal factors, and further comparative research is needed to confirm their true impact. While the absence of preserved tendon insertion and the requirement for an experienced assistant may be considered limitations, our clinical outcomes suggest that the advantages of this approach outweigh its potential drawbacks, particularly in high-risk athletic populations.

Limitations of the present study include its retrospective design, single-center setting, and the absence of a control group undergoing isolated ACL reconstruction. Additionally, the assessment of return to sport relied partly on patient-reported data, which may be subject to recall bias. Another limitation of the study is the lack of objective functional stability measures (such as instrumented laxity testing), which were not consistently available for the entire cohort and therefore not included in this analysis. Also, the absence of a standardized rehabilitation protocol could be viewed as a limitation, as patients completed their rehabilitation in different centers with potentially variable approaches. Although all patients followed the same general recommendations (immediate full weight bearing, early range-of-motion, and quadriceps activation), differences in rehabilitation intensity and supervision may have influenced functional recovery and return-to-sport outcomes. Furthermore, we did not record the exact type of sport for each patient; instead, athletes were categorized according to their Tegner activity score, which broadly reflects the level of athletic participation. This limits the possibility of sport-specific subgroup analyses. The minimum follow-up was limited to mid-term outcomes, and longer-term studies are warranted to confirm the durability of our results.

## 5. Conclusions

In summary, our modified combined ACL and ALL reconstruction technique demonstrates favorable mid-term outcomes in young and professional high-level athletes, with low rerupture rates, high return-to-sport performance rates, and no severe complications. Given its potential to protect both the ACL graft and repaired menisci, this technique should be considered in high-risk athletic populations where optimal rotational stability is critical.

## Figures and Tables

**Table 1:** Functional outcomes at final follow-up (n = 291).

Outcome	n (%) of Patients
Graft rerupture	11 (3.78%)
Return to sport at pre-injury level	220 (75.6%)
Tegner Activity Scale	
Preoperative score 10 (n = 55)	43 regained same score (78.2%)
Preoperative score 9 (n = 35)	24 regained same score (68.6%)
Preoperative score 7–8 (n = 153)	128 regained same score (83.7%)

## Data Availability

The data presented in this study is available upon request to the corresponding author. Data was not made publicly available to maintain patient privacy.
